# Price, Availability, and Youth Obesity: Evidence From Bridging the Gap

**Published:** 2009-06-15

**Authors:** Frank J. Chaloupka, Lisa M. Powell

**Affiliations:** Department of Economics and Institute for Health Research and Policy, University of Illinois at Chicago; University of Illinois at Chicago, Chicago, Illinois

## Abstract

After a decade of analyzing environmental influences on substance use and its consequences among youth in the United States, the Robert Wood Johnson Foundation's Bridging the Gap program has begun studying the effect of environmental factors on youth physical activity, diet, and weight outcomes. Much of this research has focused on access to food, as reflected by availability and price. Program researchers have documented disparities in access to healthy foods and opportunities for physical activity; healthier food outlets and opportunities for physical activity are relatively less available in communities with lower income and larger proportions of racial/ethnic minority populations. They also have found that healthier environments are associated with more fruit and vegetable consumption, more physical activity, lower body mass index, and reduced likelihood of obesity among youth.

## Introduction

The Robert Wood Johnson Foundation's Bridging the Gap program (BTG), created in 1997, has studied the effect of policies, programs, and other environmental influences on adolescent alcohol, tobacco, and illicit drug use and related outcomes in the United States (1-4). BTG is an interdisciplinary, multisite initiative that brings together a mix of original and archival data at multiple levels of social organization, including schools, communities, and states. During its first decade, BTG helped to build the evidence base on the effects of prices, control policies, and other environmental influences on youth substance use and abuse among youth; this evidence has been a key factor in the adoption of policies that have reduced youth tobacco and other substance use during this time (1). BTG focuses on youth because these behaviors largely begin during adolescence and because of the lasting effect of effective interventions to reduce initiation and uptake during adolescence.

As recognition of the obesity epidemic has increased, researchers, policy makers, and public health professionals have focused more attention on the role of environmental factors as determinants of healthy and unhealthy eating, physical activity and inactivity, and weight outcomes. BTG's research focus has turned to applying the approach it successfully used for a decade to looking at the effect of relevant environmental factors on youth physical activity, diet, and weight outcomes. The conceptual framework for BTG's research on adolescent obesity (Figure) draws on multiple disciplines and emphasizes environmental factors and their interaction with individual and social factors in affecting diet, physical activity, and weight status. Much of BTG's initial work on youth obesity has focused on access to food and physical activity outlets, as reflected by availability and price. We provide a brief overview of BTG's research on environmental factors and related implications for policies to reduce obesity and its consequences.

**Figure 1:**
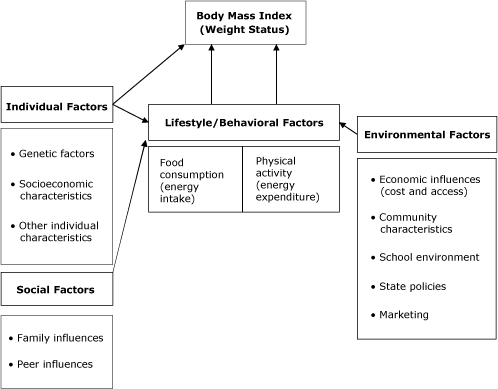
Conceptual model for Robert Wood Johnson Foundation's Bridging the Gap Program.

## Availability of Food Outlets and Opportunities for Physical Activity

BTG's earliest research on the contextual factors that contribute to overweight and obesity concentrated on documenting differences in the availability of food and physical activity-related outlets across communities (5-9). Most of this research used data from the Dun & Bradstreet MarketPlace Database, compiled in 2000 by zip code to analyze differences in availability of these outlets based on community characteristics, including racial/ethnic composition of the population and median household income. Measures of population characteristics for the 28,050 zip codes in which nearly all of the population resided in 2000 were taken from that year's census database.

Measures for the density of food-related outlets based on standard industry classification codes were developed for chain supermarkets, independent supermarkets, convenience stores, grocery stores, fast-food restaurants, and full-service restaurants. Comparable measures for the density of physical activity-related outlets were developed for membership and nonmembership health clubs, spas, and other fitness facilities; membership sports and recreation clubs; and dance studios, schools, and public dance halls. One study used measures of publicly available opportunities for physical activity, including sports areas, parks and green spaces, playgrounds, public pools and beaches, and bike paths collected around nationally representative samples of US secondary schools in 2002 and 2003 as part of BTG's extensive original data collection efforts (5).

This research consistently documented racial/ethnic and income-related disparities in the availability of healthy food and opportunities for physical activity across communities. For example, availability of free opportunities for physical activity was lower in communities with lower income levels and in those where a greater percentage of the local population was African American (5). Similar differences were observed for commercial physical activity-related outlets, which were also less available in communities with larger proportions of Latinos (6).

Fewer chain supermarkets were in lower-income communities; predominantly African American communities had approximately half as many chain supermarkets as predominantly white communities, and predominantly Latino communities had approximately one-third as many as did largely non-Latino communities (7). In contrast, smaller groceries and independent supermarkets were more available in communities with relatively larger minority populations. Previous research has shown that larger food stores (eg, supermarkets) are more likely to stock healthful foods than smaller food stores (eg, groceries and convenience stores) (10,11) and to offer foods at a lower cost (12-14). Similarly, absolute and relative (as a share of all restaurants) availability of fast-food restaurants is higher in lower- and middle-income communities than in high-income communities (8). Restaurants of all types were less available in predominantly minority communities, but a significantly higher proportion of restaurants were fast-food restaurants in these communities. Finally, fast-food restaurants and convenience stores are readily available around US secondary schools, particularly high schools; the highest availability is around schools in larger cities and lower-income neighborhoods (9).

Given the evidence that overweight and obesity are more prevalent among lower-income and minority populations, including adolescents (15,16), BTG's research highlighting community-level disparities in access to healthier food outlets and opportunities for physical activity suggests a link between access and weight outcomes. BTG's more recent research has turned to establishing relationships between access (both physical access, as reflected by availability, and economic access, as reflected by prices), healthy eating, physical activity, and obesity among adolescents.

## How Availability of Food and Opportunities for Physical Activity Influence Health Behaviors Among Adolescents

BTG's research assessing the effect of availability of food and physical activity-related outlets on adolescents' eating, physical activity, and weight outcomes links the business list data to outcomes from the annual Monitoring the Future (MTF) surveys of US secondary school students. These are nationally representative surveys of approximately 45,000-50,000 8th-, 10th-, and 12th-grade students conducted each spring in approximately 420 schools by the University of Michigan's Institute for Social Research (17). These surveys are a key source of information on adolescent tobacco, alcohol, and illicit drug use in the United States, and they yield data on related attitudes, beliefs, and perceived access, as well as key socioeconomic and demographic information.

Since 1986, the MTF surveys have also collected information on self-reported height and weight, which can be used to calculate body mass index (BMI) and indicators for youth obesity. These surveys also collect information on dietary habits (eg, frequency of eating fresh fruit and vegetables), physical activity (eg, frequency of exercise, vigorous exercise and sports participation, including participation on a school team), sedentary behavior (eg, frequency of television watching and leisure time computer use), and other behaviors potentially related to weight outcomes (eg, sleep patterns). Consistent with available data from other nationally representative surveys such as the National Health and Nutrition Examination Survey (18,19) and the Youth Risk Behavior Surveillance System (20), data from the MTF surveys show generally increasing BMI and prevalence of obesity among US adolescents, coupled with rising screen time and reductions in physical activity, healthy eating, and sleep (21,22).

### Effect on adolescent physical activity

Data from 1997 through 2003 on the availability of commercial physical activity-related outlets (fitness facilities, membership sports and recreation clubs, and dance studios, halls, and schools) have been linked to 8th-, 10th-, and 12th-grade student self-reports of physical activity (frequency of participation in sports/athletics and exercise) (23). Outlet density measures were matched by zip code, based on the zip code of each school participating in the MTF study during these years. Living in a community with greater availability of commercial physical activity outlets was associated with increased reports of frequent participation in sports and exercise, and larger associations were observed for girls and for older students (12th graders). Simulations conducted using these estimates implied, for example, that, for 12th-grade girls, living in a community with a relatively high availability of these outlets (8 facilities) was associated with a 9% higher prevalence of frequent vigorous exercise than in a community with limited availability (1 facility); for 12th-grade boys, the comparable higher prevalence was 6%.

### Effect on adolescent healthy eating and weight

Using 8th- and 10th-grade students' self-reported height and weight to calculate BMI and an indicator for obesity, researchers (24) examined the associations between food store availability and adolescent weight outcomes for the years 1997 through 2003. Outlet density measures for supermarkets (chain and independent), grocery stores, and convenience stores were matched to the MTF survey data based on the zip code of participating schools. Differential effects are expected by store type, given previous research on the relationship between store type and availability and prices for healthier foods (10-14). Indeed, the results from the MTF study show that more availability of chain supermarkets was associated with lower BMI and reduced likelihood of obesity among adolescents, whereas more availability of convenience stores was associated with higher BMI and higher probability of being obese. Both associations were stronger for adolescents in households where their mother worked full-time, and the association for chain supermarkets was stronger for African American adolescents.

In contrast, researchers found little association between fast-food and full-service restaurant availability and adolescent diet or weight outcomes (25). Using similarly calculated measures of restaurant outlet density matched to the MTF survey data based on the zip codes of participating schools, they found no significant associations between either fast-food or full-service restaurant availability and any of the outcomes examined, except for a small positive association between the availability of full-service restaurants and frequency of fruit and vegetable consumption.

## How Food Prices Influence Eating Behavior and Weight Among Adolescents

BTG researchers have similarly examined the associations between food prices and adolescent diet and weight outcomes by using data from the American Chamber of Commerce Research Association (ACCRA) quarterly cost-of-living index reports matched to the MTF survey data. The ACCRA reports include prices for goods and services, including 3 fast foods (McDonald's Quarter Pounder with cheese, Pizza Hut or Pizza Inn pizza, and a KFC or Church's Chicken chicken meal) and 7 fruits and vegetables (bananas, canned peaches, canned sweet peas, canned tomatoes, lettuce, potatoes, and frozen corn). BTG researchers used these price data to create 2 price indices, 1 for fast-food prices (a simple average of the 3 prices) and 1 for fruits and vegetables (a weighted average based on household expenditure shares). ACCRA (now the Council for Community and Economic Research) collects prices for about 300 metropolitan statistical areas each quarter; prices were matched to MTF schools based on the prices in the metropolitan statistical area nearest each school.

Researchers used MTF 1997-2003 data on 8th- and 10th-grade students to analyze the effect of prices on frequency of fruit and vegetable consumption, BMI, and an indicator for obesity (25). As expected given economic theory, they found that lower prices for fruits and vegetables were associated with more fruit and vegetable consumption, while lower prices for fast foods were associated with less fruit and vegetable consumption. Consistent with the evidence that healthier eating results in better weight outcomes, they found that lower fruit and vegetable prices were associated with lower BMI, and lower fast-food prices were associated with higher BMI. Finally, they found that lower fast-food prices were also associated with an increased likelihood of obesity. Their estimates imply that a 10% increase in fast-food prices would raise the probability of frequent fruit and vegetable consumption by 3%, reduce BMI by 0.4%, and lower the likelihood of obesity by 5.9% among youth.

A subsequent BTG study (26) looked at the differential effect of prices on youth at different BMI levels and explored whether prices had more or less of an effect on youth at increased risk for obesity (those at higher BMIs). Using quantile regression methods applied to the same data, researchers found that associations between fast-food and fruit and vegetable prices were strongest for youth at the upper end of the BMI distribution. For example, they found that the association between fast-food prices and BMI was approximately 4 times greater for youth above the 90th percentile than it was for the overall sample of youth, implying that a 10% increase in fast-food prices would lower BMI by 10% to 11% among youth who weighed above the 90th percentile. Similarly, the association between fruit and vegetable prices and BMI was approximately 5 times greater for youth who weighed above the 95th percentile than for the overall sample of youth. Estimates implied that a 10% drop in fruit and vegetable prices would lower BMI by 5% to 6% in this group.

## Discussion

BTG researchers have documented that healthier food outlets and opportunities for physical activity are less available in communities with lower incomes and larger proportions of racial/ethnic minorities. These findings for youth across the nation are consistent with observations in studies that focused on 1 or a few communities. Likewise, BTG researchers have begun to demonstrate that healthier environments are associated with more fruit and vegetable consumption, more activity, lower BMI, and reduced likelihood of obesity.

These studies, however, have several limitations. For example, some studies have focused on food and physical activity environments only and have not taken into account the variety and range of environments to which a person is exposed. Moreover, because people of different ages have different interactions with their environments, these studies of youth have produced findings that do not translate to all age groups. In addition, these studies are cross-sectional, demonstrating associations and not causation. Also, the business list data and price data used to assess physical and economic availability may be subject to some measurement errors. Finally, these studies used BMI and obesity measures based on self-reported height and weight, which may be inaccurately reported (27,28).

The empirical findings presented here (based on US data) and similar study findings (based on data from other countries) have several implications for policies that could be used to curb the rising obesity epidemic. Governments can use a variety of policies to promote healthier environments, from zoning and land use regulations to fiscal policies. One of the fiscal tools that governments can use to promote development of new supermarkets, fitness facilities, and others is to provide tax incentives to businesses that are considering opening facilities in underserved communities; several have begun to do this to attract supermarkets to "food deserts" — areas with little or no ready access to relatively healthy foods. Similarly, governments can directly provide opportunities for physical activity by investing in local recreation centers, parks, and other resources in underserved areas.

Governments also can use taxes and subsidies to alter the relative prices of foods and beverages. Most food subsidy programs to date aim to improve food security by subsidizing food purchases by low-income households (eg, food stamps) and have few restrictions on what products can be purchased. Subsidies that target healthy foods would reduce prices for these products relative to those of less healthy foods and likely result in healthier eating. Similarly, governments could tax less healthy foods and beverages to raise the relative prices of these products and discourage their consumption. Forty states already impose modest sales taxes on at least 1 soft drink, candy, or snack item (29). Higher taxes that raise the prices of less healthy products would likely reduce the consumption of these products and improved weight outcomes. In addition to promoting healthier eating, such taxes could generate new revenues that could be used to support other efforts to promote healthy eating and increased physical activity, such as subsidizing healthier food products, investing in opportunities for physical activity, and supporting mass-media campaigns to promote healthy eating and increased activity.

As governments adopt policies such as taxes on less healthy products and subsidies for healthier products, investments in food and physical activity outlets, research on the effect of these policies will be needed to support future, evidence-based efforts to curb the obesity epidemic. Particularly useful will be longitudinal studies that assess the effect of these policy changes on people's behavior and on changes in weight outcomes.
